# Effectiveness of exercise interventions for chronic low back pain among office workers: a systematic review and meta-analysis

**DOI:** 10.1093/joccuh/uiag058

**Published:** 2026-09-23

**Authors:** Hiroaki Yamano, Ryunosuke Urata, Kimihiro Ogawa, Takuya Hatade, Ryuichi Shimada, Hiroki Yokoyama, Hikaru Tanaka, Kazuaki Mori, Shinsuke Suetake

**Affiliations:** Department of Physical Therapy, Faculty of Health Care Sciences, Takarazuka University of Medical and Health Care, 1 Hanayashiki-Midorigaoka, Takarazuka, Hyogo 666-0162, Japan; New Spine Clinic Tokyo, Tokyo, Japan; AViC THE PHYSIO STUDIO, TOYOTA TSUSHO ALL LIFE Corporation, Tokyo, Japan; Geriatric Health Services Facility “Hidamari-no-sato,” Amagasaki Health and Welfare Cooperative, Hyogo, Japan; Department of Occupational Therapy, Faculty of Health Sciences, Iryo Sosei University, Fukushima, Japan; Department of Rehabilitation, Kansai Medical University Kuzuha Hospital, Osaka, Japan; Journey Rehab Co, Ltd, Chiba, Japan; Department of Rehabilitation Medicine, Nishikagawa Municipal Hospital, Kagawa, Japan; Caname Inc, Tokyo, Japan

**Keywords:** low back pain, exercise therapy, office workers, occupational health, systematic review, meta-analysis

## Abstract

**Objectives:**

Chronic low back pain (LBP), largely attributable to prolonged sitting and low levels of physical activity, is prevalent among office workers and constitutes a major occupational health concern. Although exercise therapy is widely recommended for chronic LBP, limited studies specifically target office workers. In this study, we systematically evaluated the effectiveness of exercise interventions for office workers with chronic LBP, focusing on pain intensity, functional disability, physical activity level, and work-related outcomes.

**Methods:**

A systematic review and meta-analysis of randomized controlled trials (RCTs) was conducted. Electronic databases were searched for RCTs published through March 31, 2025. Primary outcomes were pain intensity and work productivity; secondary outcomes were functional disability and physical activity level. Meta-analyses were performed using a random-effects model, and the certainty of evidence was assessed using the Grading of Recommendations Assessment, Development, and Evaluation approach.

**Results:**

We included 4 RCTs, involving 263 participants, in this review. At short-term follow-up, the pooled estimate suggested that exercise interventions may reduce pain intensity compared with control conditions. However, the certainty of evidence was very low, and evidence for functional disability and physical activity level was insufficient. None of the included studies assessed work productivity or presenteeism using validated instruments. The certainty of evidence for pain and functional disability outcomes was rated as very low.

**Conclusions:**

Exercise interventions may reduce short-term pain in office workers with chronic LBP; however, evidence regarding functional and work-related outcomes remains insufficient. High-quality, long-term studies incorporating work productivity and presenteeism as primary outcomes are required.

## Teaser

**Key points**
 

**What is already known on this topic**:
  - Exercise therapy is widely recommended as a nonpharmacological treatment for chronic low back pain.
  - Evidence regarding the effectiveness of exercise therapy for reducing pain and improving function in office workers is limited.
  **What this study adds**:
  - Exercise interventions may reduce short-term pain intensity among office workers with chronic LBP, although the certainty of evidence is very low.
  **How this study might affect research, practice, or policy**:
  - This review suggests that exercise therapy may be considered for short-term pain management in occupational health practice, while highlighting the limited certainty of the current evidence.
  - Occupationally relevant outcomes should be combined with clinical measures when evaluating interventions for chronic LBP in workplace settings.

## 1. Introduction

Chronic low back pain (LBP) is among the most prevalent musculoskeletal disorders worldwide and a leading contributor to disease burden, as measured by disability-adjusted life years.^1^^,^^2^ In addition to reducing quality of life, LBP imposes a substantial socioeconomic burden through decreased work productivity and increased health care costs.^3^ Among office workers, prolonged sitting and low levels of physical activity are major risk factors for LBP.^4^ More recently, the widespread adoption of teleworking and virtual meetings during the COVID-19 pandemic further increased sedentary time and reduced opportunities for physical activity, thereby elevating the risk of LBP onset and chronicity.^5^ Chronic LBP among office workers is more strongly associated with productivity losses due to presenteeism than those due to absenteeism, resulting in considerable economic losses for organizations and society.^6^ Consequently, chronic LBP in sedentary work environments represents a critical occupational health concern requiring effective preventive and therapeutic strategies.

Exercise therapy is widely recommended as a nonpharmacological treatment for chronic LBP, and numerous randomized controlled trials (RCTs) and systematic reviews have reported beneficial effects on pain and physical function.^7–9^ However, the applicability of this evidence to office workers remains uncertain, because many previous reviews have primarily focused on clinical populations recruited from health care settings or on broad worker populations without specifically addressing the occupational characteristics of sedentary office work.

Several systematic reviews have examined work-related interventions for LBP or musculoskeletal disorders in workers. Ishimaru et al.^10^ reviewed fitness-for-work interventions for workers with LBP and evaluated occupationally relevant outcomes, including return to work, sick leave, pain intensity, disability, and work ability. However, their review focused on workers in general and included interventions based on expert assessment, workplace advice, and work modification, rather than exercise-based interventions specifically designed for sedentary office workers. Russo et al.^11^ evaluated workplace interventions for workers with LBP and reported improvements in pain and disability; however, the included interventions were heterogeneous and comprised technical, educational, ergonomic, behavioral, and exercise components. Therefore, the specific effect of exercise-based interventions in office workers with chronic LBP remains unclear. More recently, Tersa-Miralles et al.^12^ reviewed workplace exercise interventions for musculoskeletal disorders among office workers and suggested that such interventions may reduce musculoskeletal pain. Nevertheless, that review included musculoskeletal disorders across multiple body regions and was not limited to chronic LBP. In addition, Eisele-Metzger et al.^13^ conducted a systematic review and network meta-analysis of interventions for preventing back pain among office workers. Although this review provided important evidence regarding preventive interventions in office settings, it primarily addressed prevention of back pain, including pain in any spinal region, rather than treatment of chronic LBP in symptomatic office workers.

Thus, despite the growing body of evidence on exercise therapy for chronic LBP and workplace-based interventions for workers, an evidence gap remains regarding randomized exercise interventions for office workers with chronic LBP. Furthermore, although LBP is closely related to occupational outcomes such as work performance, sickness absence, and presenteeism, these outcomes have not been consistently examined in previous exercise-focused reviews. Therefore, the present review specifically focused on RCTs of exercise-based interventions for office workers with chronic LBP. The aim of this study was to quantitatively evaluate the effectiveness of exercise interventions for office workers with chronic LBP through a systematic review and meta-analysis of RCTs. Specifically, we assessed the effects of exercise interventions on pain intensity and functional disability. In addition, we examined whether occupationally relevant outcomes, including work productivity and presenteeism, were assessed in the included studies. This approach allowed us to describe not only the effects of exercise interventions on clinical outcomes but also the extent to which work-related outcomes have been considered in this research area.

## 2. Materials and methods

### 2.1 Study design and protocol registration

In this systematic review and meta-analysis, we evaluated the effectiveness of exercise interventions for office workers with chronic LBP. The study protocol was prospectively registered in PROSPERO before study selection and data extraction were undertaken; the registration was published on June 12, 2023 (registration number: CRD42023431543).^14^ The review was conducted and reported in accordance with PRISMA 2020 guidelines.^15^ Only RCTs were included, and meta-analyses were performed to synthesize the intervention effects. The certainty of evidence for primary and secondary outcomes was assessed using the Grading of Recommendations Assessment, Development, and Evaluation (GRADE) approach.^16^

### 2.2 Eligibility criteria

We included RCTs evaluating exercise interventions for office workers with chronic LBP. The eligibility criteria were as follows. (1) Participants were office workers aged ≥18 years with chronic LBP. Chronic LBP was primarily defined as pain persisting for ≥3 months. Participants were required to report a pain score of ≥1 on the visual analog scale (VAS), numerical rating scale, or Likert scale or a self-reported presence of LBP based on questionnaires or interviews.^17^ Office workers were defined as individuals engaged primarily in desk-based or sedentary work. Studies were included without further assessment when the target population was explicitly described as “office workers,” “desk workers,” or an equivalent term. For studies that did not explicitly use these terms, eligibility was determined using the following operational criteria: participants were considered office workers if they spent approximately 50% or more of their working hours in a seated position.^18^ When sitting time was not directly reported, 2 independent reviewers applied this criterion during the study selection process based on descriptions of job title, workplace setting, desk-based tasks, computer use, or other sedentary work characteristics. Studies were included only when the participant population was judged by both reviewers to be consistent with predominantly sedentary office work. (2) Interventions must have included exercise-based programs, such as individual or group exercise instruction, remotely delivered exercise (eg, via applications), physiotherapy, Pilates, yoga, tai chi, resistance training, or walking. (3) Control groups must have included either no intervention, usual care, or non–exercise-based treatment programs. (4) Only original articles or pilot studies published in Japanese or English were considered eligible for inclusion.

The exclusion criteria were as follows: nonrandomized, observational, or case–control studies; conference abstracts; studies published in languages other than Japanese or English; and studies in which the intervention consisted solely of non–exercise therapies (eg, massage, manual therapy, electrical stimulation, hydrotherapy, or ultrasound therapy).

### 2.3 Information sources and search strategy

Electronic literature searches were conducted in MEDLINE via PubMed, the Cochrane Central Register of Controlled Trials, CINAHL, Web of Science, and Ichushi-Web. The final search was performed on March 31, 2025. Search strategies combined keywords and controlled vocabulary related to LBP, exercise, and RCTs and were adapted for each database. The search term “office workers” was intentionally not included in the search strategy because it could have retrieved a substantial number of studies unrelated to the scope of this review, particularly from fields outside medicine, rehabilitation, nursing, welfare, and health care. Given that this review focused on individuals with LBP rather than a specific occupational group, the search strategy was designed to prioritize terms related to the target condition rather than occupation. Table 1 presents the detailed PubMed search strategy, and Table S1 presents the strategies for the other databases. Reference lists of eligible studies and relevant reviews were manually screened to identify additional studies. All search and study selection processes followed PRISMA guidelines.^15^

**Table 1:** Search strategy used in MEDLINE via PubMed.

(“Back Pain”[MeSH Terms] OR “back pain”[All Fields] OR backach*[All Fields] OR “back pain without radiation”[All Fields] OR “low back pain”[All Fields] OR lumbago[All Fields] OR “lower back pain”[All Fields] OR LBP[All Fields] OR “Low Back Ache”[All Fields]) AND (“Exercise”[MeSH Terms] OR exercis*[All Fields] OR “exercise therap*”[All Fields] OR “physical activit*”[All Fields] OR “muscle stretching exercis*”[All Fields] OR “physical conditioning”[All Fields] OR training[All Fields] OR walking[All Fields] OR stretch*[All Fields] OR strengthening[All Fields] OR “muscle strength”[All Fields] OR “Physical Therapy Modalities”[MeSH Terms] OR “physical therapy”[All Fields] OR physiotherapy[All Fields] OR manipulation*[All Fields] OR “workplace exercise intervention”[All Fields] OR practice[All Fields] OR mobilization[All Fields]) AND (randomized[Title/Abstract] OR randomised [Title/Abstract] OR randomly[Title/Abstract])
Number of records: 6007 Date: March 31, 2025

### 2.4 Study selection

Study selection was conducted in 2 stages. After removing duplicate records, 2 independent reviewers screened titles and abstracts and classified studies as “eligible,” “ineligible,” or “potentially eligible.” The full texts of studies classified as “eligible” or “potentially eligible” were then independently assessed by the same reviewers using predefined eligibility and exclusion criteria. Disagreements were resolved through discussion with a third reviewer. The study selection process and final number of included studies are presented in a PRISMA flow diagram (Figure 1).^15^

**Figure 1:**
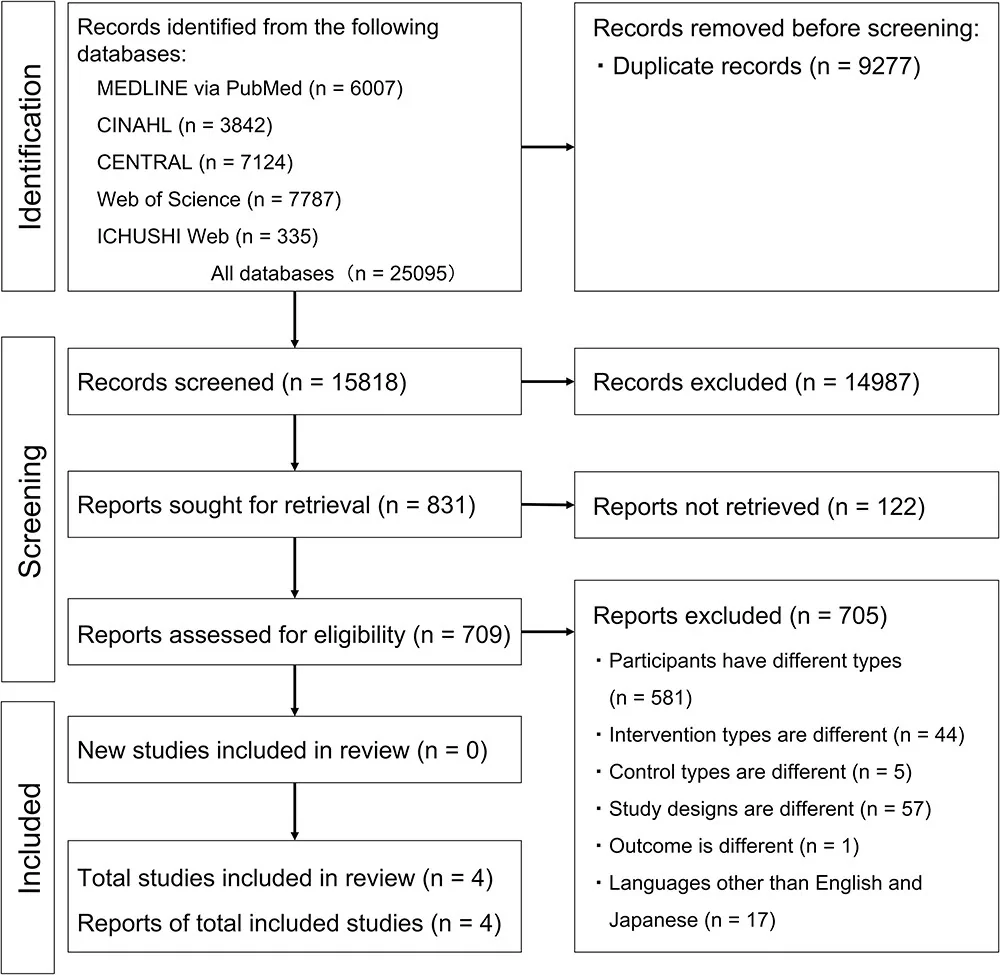
PRISMA flow diagram of the study selection process.

### 2.5 Data extraction

Two reviewers independently extracted the data using a standardized form. Extracted variables included study design, participant characteristics (age, sex, sample size, and LBP duration), intervention characteristics (exercise type, delivery format, frequency, and intervention duration), and comparator details. For primary and secondary outcomes, information on measurement instruments, assessment time points, and group-specific means and SDs was extracted. When necessary data were missing, the corresponding authors were contacted by email; if no response was received within 3 months, the data were considered missing. Disagreements were resolved through consultation with a third reviewer.

### 2.6 Outcomes

The primary outcomes were pain intensity and work productivity. Pain intensity was assessed using the VAS, numerical rating scale, Likert scale, or equivalent pain scales used in the included studies. Work productivity was defined as outcomes assessed using validated instruments, such as the Work Productivity and Activity Impairment Questionnaire, the Health and Work Performance Questionnaire, and the Work Limitations Questionnaire.^19^

The secondary outcomes were functional disability and physical activity level. Functional disability was assessed using the Oswestry Disability Index (ODI) or Roland-Morris Disability Questionnaire (RMDQ).^20^^,^^21^ Physical activity was assessed using accelerometer-based measures or the International Physical Activity Questionnaire.^22^ All outcomes were categorized by follow-up duration as either short-term (<3 months post-intervention), mid-term (3-6 months), or long-term (≥12 months).

### 2.7 Risk-of-bias assessment

Risk of bias was assessed using the revised Cochrane risk-of-bias tool for randomized trials (RoB 2).^23^ The following domains were evaluated: bias arising from the randomization process, bias due to deviations from intended interventions, bias due to missing outcome data, bias in measurement of the outcome, and bias in selection of the reported result. Two reviewers independently assessed each included study, and disagreements were resolved through discussion with a third reviewer. The overall risk-of-bias judgment for each study was classified as low risk, some concerns, or high risk according to RoB 2 guidance.^23^

### 2.8 Data synthesis and meta-analysis

Meta-analysis was conducted when at least 2 studies reported the same or sufficiently comparable outcomes at a similar follow-up time point and when the outcome construct, scale direction, and effect measure allowed meaningful quantitative synthesis. When the same outcome construct was assessed using different measurement scales, the results were converted to a common metric prior to pooling. When outcome measures, intervention duration, or comparator conditions were judged to be too heterogeneous for statistical pooling, the findings were summarized narratively.

Statistical analyses and meta-analyses were conducted using Review Manager version 5.3.^24^ A random-effects model was used for meta-analysis. For continuous outcomes, mean differences (MDs) or standardized mean differences (SMDs) with 95% CIs were calculated. SMDs were used when the studies employed different outcome scales. Statistical heterogeneity was assessed using the I^2^ statistic, with values ≥50% indicating substantial heterogeneity. When only medians and interquartile ranges were reported, means and SDs were estimated using the method proposed by Wan et al.^25^

### 2.9 Sensitivity analysis

Additional sensitivity analyses not included in the original protocol were conducted to investigate the causes of substantial heterogeneity in the results and to assess the robustness of integrated data. Sensitivity analyses were performed by excluding studies with the most heterogeneous outcome measure regarding pain intensity and those with a high risk of bias.

### 2.10 Certainty-of-evidence assessment

The certainty of evidence for all primary and secondary outcomes was assessed using the GRADE approach.^16^ Evidence was evaluated across 5 domains—risk-of-bias, indirectness, inconsistency, imprecision, and publication bias—and rated as high, moderate, low, or very low certainty.

## 3. Results

### 3.1 Study characteristics

The literature search identified 25 095 records. After removing duplicates and screening titles, abstracts, and full texts, 4 RCTs were ultimately included in this review.^26–29^ The study selection process and number of included studies are presented in the PRISMA flow diagram (Figure 1).^15^

A total of 263 participants were included in the studies, with a predominance of female participants. Excluding 1 study with missing demographic data,^26^ the review included 45 males and 132 females. All participants had LBP that had persisted for at least 3 months. All included studies implemented exercise programs in the intervention groups. Exercise interventions varied and included supervised individual exercise programs, workplace-based group exercise programs, home-based self-exercises, web-based exercise instructions, and smartphone application-based exercise guidance. Intervention duration ranged from 6 weeks to 8 weeks. Table S2 summarizes the detailed characteristics of all included studies.

### 3.2 Risk-of-bias assessment

Figure 2 presents the RoB 2 assessment. One study was judged to pose a low risk, 1 study raised some concerns, and 2 studies were judged to pose a high risk. Overall, there were concerns about the potential risk of bias in the measurement of the outcome and the selection of the reported results. The results for each outcome are shown in Table S3.

**Figure 2:**
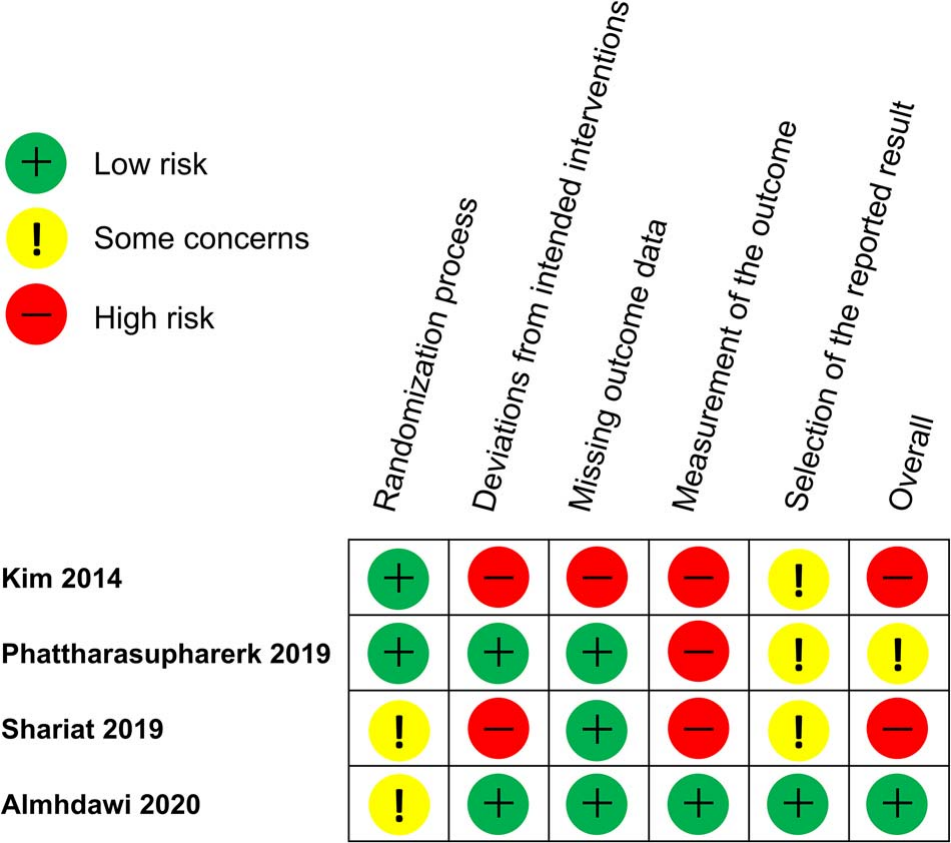
Risk-of-bias assessment of the included studies.

### 3.3 Effects of interventions

#### 3.3.1 Pain intensity

Four studies reported short-term outcomes for pain intensity. After detailed review of the studies, 3 studies used the VAS,^27–29^ whereas 1 study used the Functional Rating Index.^26^ Among the 3 studies using the VAS, 2 studies^27^^,^^29^ employed a 0-100 scale and 1 study^28^ employed a 0-10 scale. To harmonize the outcome measures across studies, the mean values and SDs reported on the 0-10 scale were multiplied by 10 and converted to a 0-100 scale. This linear transformation preserves the relative differences, variance structure, and effect estimates while enabling direct comparison across studies using different score ranges.

Although the pooled analysis of all 4 studies demonstrated significantly greater pain reduction in the intervention group than in the control group, substantial heterogeneity was observed (*n* = 202; SMD = −3.34; 95% CI, −5.25 to −1.43; *P* < .001; I^2^ = 95%) (Figure 3A). Sensitivity analysis excluding the study that used the Functional Rating Index yielded a significant pain-reducing effect in the intervention group and markedly reduced heterogeneity (*n* = 166; MD = −39.02; 95% CI, −44.18 to −33.86; *P* < .001; I^2^ = 26%) (Figure 3B). Furthermore, although 1 study was judged to have a high risk of bias,^29^ sensitivity analyses showed that excluding this study did not alter the conclusions or the certainty of the evidence (*n* = 113; MD = −33.89; 95% CI, −42.20 to –25.58; *P* < .001, I^2^ = 0%).

**Figure 3:**
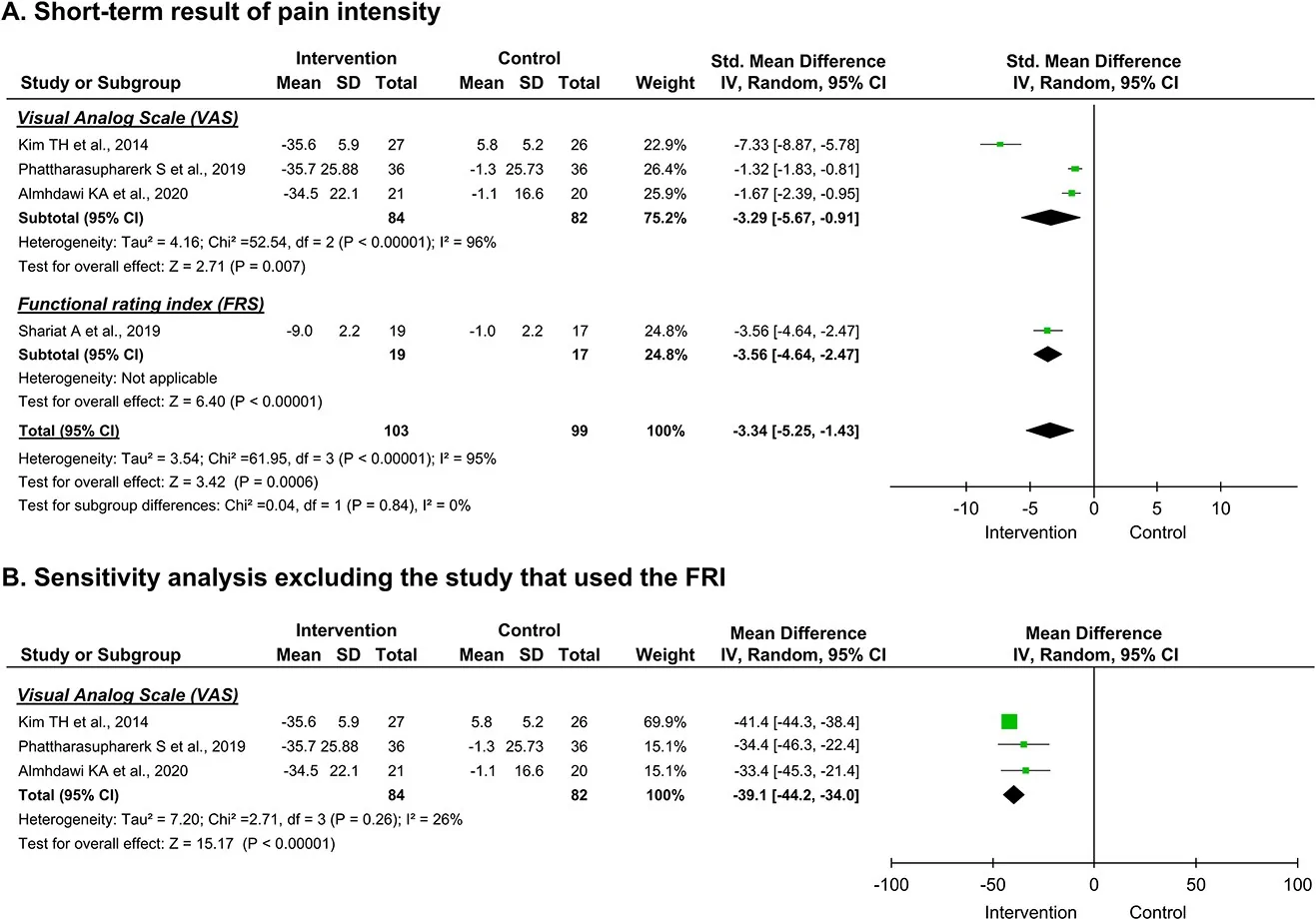
Forest plots of short-term pain intensity outcomes.

No studies reported mid-term or long-term outcomes; therefore, meta-analyses were not performed for these time points.

#### 3.3.2 Work productivity

None of the included studies assessed work productivity outcomes. Specifically, no study evaluated work productivity or presenteeism using validated instruments such as the Work Productivity and Activity Impairment Questionnaire, the Health and Work Performance Questionnaire, or the Work Limitations Questionnaire.^19^ Accordingly, no meta-analyses of work productivity outcomes were performed.

#### 3.3.3 Functional disability

Two studies reported short-term outcomes for functional disability. After a detailed review of the outcome measures, one study used the RMDQ,^27^ whereas the other used the ODI.^28^ In the study using the RMDQ, functional disability improved significantly more in the intervention group than in the control group (*n* = 72; MD = −2.00; 95% CI, −3.57 to –0.43, *P* = .02).^27^ Similarly, the study using the ODI reported significantly greater improvement in functional disability in the intervention group than in the control group (*n* = 41; MD = −10.47; 95% CI, −16.57 to −4.37, *P* = .002).^28^

Although both studies assessed functional disability, a meta-analysis was not conducted because different outcome measures were used and the outcomes were considered insufficiently comparable for quantitative synthesis. No studies reported mid-term or long-term outcomes; therefore, meta-analyses were not conducted for these periods.

#### 3.3.4 Physical activity

Physical activity level was assessed using the International Physical Activity Questionnaire in only 1 study.^22^^,^^28^ The reported MD refers to total physical activity expressed as MET-minutes/week. Consequently, a meta-analysis of physical activity outcomes was not performed. Based on this single study, we observed no significant between-group difference in the short term (*n* = 41; MD = −1635.85 MET-minutes/week; 95% CI, −3876.07 to 604.37; *P* = .168).

No studies reported mid-term or long-term outcomes; therefore, meta-analyses were not performed for these time points.

### 3.4 Certainty of evidence

Table S4 summarizes the certainty of evidence. All outcomes were downgraded by 1 level because publication bias could not be ruled out.^30^ The evidence for short-term pain intensity was further downgraded by 1 level for risk of bias and 1 level for imprecision. The evidence for short-term functional disability was downgraded by 1 level for risk of bias and 2 levels for imprecision, whereas the evidence for short-term physical activity was downgraded by 1 level for inconsistency and 2 levels for imprecision. Consequently, all outcomes were rated as very low-certainty evidence.

## 4. Discussion

The main contribution of this review is that it synthesizes randomized evidence on exercise interventions specifically for office workers with chronic LBP and identifies an important gap in the assessment of work productivity and presenteeism. The pooled estimate suggested that exercise interventions may reduce pain intensity in the short term among office workers with chronic LBP; however, the certainty of evidence was very low. Evidence for functional disability, physical activity level, work productivity, and presenteeism remains insufficient. Notably, none of the included studies assessed work productivity or presenteeism using validated instruments. By extending the existing evidence on exercise therapy for chronic LBP from health care–based patient populations to occupational settings, this study highlights critical gaps regarding functional and productivity-related outcomes among office workers.^7–9^^,^^19^

The observed short-term pain reduction associated with exercise interventions is consistent with previous systematic reviews and meta-analyses conducted in broader populations with chronic LBP.^7–9^ Exercise therapies—including strengthening, aerobic exercise, and stretching—have been shown to produce moderate pain relief, with effects that appear relatively independent of exercise modality.^8^^,^^9^ Despite substantial heterogeneity in intervention content, the included studies generally favored exercise interventions for short-term pain reduction. Several mechanisms may plausibly explain the observed short-term pain reduction, although these mechanisms were not directly tested in the included studies. Exercise may improve trunk and hip muscle function, joint mobility, confidence in movement, and short-term physical activity exposure, all of which may be relevant for office workers exposed to prolonged sitting. However, these explanations remain speculative and should be examined in future studies that include mechanistic outcomes.^31^^,^^32^ Additionally, exercise interventions that can be implemented in the workplace or at home may be easier to integrate into daily routines than clinic-based interventions, potentially improving short-term adherence and contributing to pain reduction.^33^

The included interventions differed substantially in exercise modality, delivery format, supervision, duration, and comparator conditions. In response to this heterogeneity, we conducted an additional analysis restricted to studies using the VAS. The reduction in statistical heterogeneity from I^2^ = 95% in the initial pooled analysis to I^2^ = 26% in the VAS-only analysis suggests that differences in outcome measures contributed substantially to the observed heterogeneity. However, this finding does not eliminate concerns regarding clinical heterogeneity, including differences in exercise modality, intervention duration, supervision, and comparator conditions. Therefore, the pooled estimate should be interpreted as evidence of a possible overall effect of exercise-based interventions rather than as evidence supporting any specific exercise protocol. The present review cannot determine which specific type, dose, or delivery format of exercise is most effective for office workers with chronic LBP.

Evidence regarding functional disability and physical activity was limited. Although the individual studies that assessed functional disability reported greater improvements in the intervention groups, a meta-analysis was not conducted because different outcome measures were used and only 2 studies were available. Thus, the current evidence does not allow firm conclusions regarding the effect of exercise interventions on functional disability in office workers with chronic LBP. One possible explanation is that office workers who remain employed may have relatively mild baseline disability, which may limit the responsiveness of measures such as the ODI and RMDQ.^20^^,^^21^^,^^34^ In addition, improvements in disability and daily activity may require longer intervention periods and sustained behavioral change, whereas the included studies evaluated only short-term outcomes. Evidence for physical activity level was also insufficient because only 1 study assessed this outcome, and the assessment relied on self-reported measures, which is susceptible to measurement error and reporting bias.^22^

From an occupational health perspective, this review highlights a lack of studies evaluating work productivity or presenteeism. Chronic LBP imposes substantial economic costs, primarily through presenteeism rather than absenteeism.^6^^,^^19^^,^^35^ Nevertheless, exercise intervention studies in occupational settings have largely focused on clinical outcomes, such as pain and functional disability, while neglecting work-related outcomes that are highly relevant to both office workers and employers. This discrepancy may reflect the tendency of intervention studies to prioritize clinical outcomes over occupationally relevant outcomes. For office workers with chronic LBP, improvements in work performance, concentration, and task endurance may be more practically meaningful than pain reduction alone.^36^ The findings of this review suggest that future intervention studies should incorporate work productivity and presenteeism as core outcomes alongside clinical measures.

This study has some limitations. First, the number of included RCTs was small, and sample sizes were relatively limited, contributing to imprecision in effect estimates and very low certainty of evidence. Second, risk-of-bias concerns should be considered when interpreting these findings. Because blinding of participants and intervention providers is generally difficult in exercise trials, deviations from intended interventions and expectation effects may have influenced subjective outcomes such as pain intensity and self-reported functional disability. Third, only short-term outcomes were available, preventing an assessment of the durability of intervention effects. Fourth, the definition of office workers may have introduced some misclassification. Although we operationalized office workers as individuals engaged in predominantly sedentary work, not all included studies explicitly quantified occupational sitting time. Therefore, eligibility was partly inferred from study descriptions of job type and work setting, which may limit the precision and generalizability of the findings. Fifth, heterogeneity in intervention content, outcome measures, and comparator conditions may limit generalizability. Finally, restricting the literature search to Japanese and English publications may have introduced language bias and excluded relevant unpublished studies. Future research should focus on well-defined office worker populations with chronic LBP, employ larger sample sizes, and incorporate long-term follow-ups with work productivity and presenteeism as primary outcomes. Evaluating adherence, adverse events, feasibility, and cost-effectiveness would further enhance the applicability of the results to occupational health practice.

## 5. Conclusions

Exercise interventions may reduce short-term pain intensity among office workers with chronic LBP; however, the certainty of evidence was very low. The findings of this systematic review and meta-analysis suggest that exercise interventions may be beneficial for pain management in this population, but their effects on work-related and functional outcomes cannot be determined conclusively from current evidence. Future large-scale, long-term RCTs should clearly define office worker populations and include work productivity and presenteeism as primary outcomes. Overall, this study provides preliminary evidence highlighting the importance of integrating occupationally relevant outcomes with clinical measures when evaluating interventions for chronic LBP in workplace settings.

## Supplementary Material

modified_supplemental_material_20260918_uiag058

## Data Availability

No new data were generated or analyzed in support of this research.
