# Supplementary material for: Effectiveness of exercise interventions for chronic low back pain among office workers: a systematic review and meta-analysis
Source: J Occup Health. 2026 Sep 23;68(1):uiag058. doi: 10.1093/joccuh/uiag058 (PMC13695462; doi:10.1093/joccuh/uiag058)
Supplement: Supplementary file 1 [file modified_supplemental_material_20260918_uiag058.docx]

**Supplementary Table 1.** Detailed search strategies for each electronic database

| **Cochrane Central Register of Controlled Trials** | |
| --- | --- |
| #1 | [mh “back pain”] |
| #2 | "back pain" |
| #3 | “back pain without radiation” |
| #4 | “low back pain” |
| #5 | lumbago |
| #6 | “lower back pain” |
| #7 | “LBP” |
| #8 | "Low Back Ache" |
| #9 | {OR #1-8} |
| #10 | [mh exercise] |
| #11 | exercis* |
| #12 | “exercise therap*" |
| #13 | "physical activit*" |
| #14 | "muscle stretching exercis*" |
| #15 | "physical conditioning" |
| #16 | training |
| #17 | walking |
| #18 | stretch* |
| #19 | strengthening |
| #20 | "muscle strength" |
| #21 | [mh "physical therapy modalities"] |
| #22 | "physical therapy" |
| #23 | physiotherapy |
| #24 | manipulation* |
| #25 | "Workplace exercise intervention" |
| #26 | "practice" |
| #27 | "mobilization" |
| #28 | {OR #10-27} |
| #29 | randomized:ti,ab |
| #30 | randomised:ti,ab |
| #31 | randomly:ti,ab |
| #32 | {OR #29-31} |
| #33 | #9 AND #28 AND #32 |
|  | Number of records: 7124  Date: March 31, 2025 |

| **CINAHL** | |
| --- | --- |
| S1 | MH back pain |
| S2 | TX back pain |
| S3 | TX backach* |
| S4 | TX back pain without radiation |
| S5 | TX low back pain |
| S6 | TX lumbago |
| S7 | TX lower back pain |
| S8 | TX LBP |
| S9 | TX Low Back Ache |
| S10 | S1 OR ~ S9 |
| S11 | MH exercise |
| S12 | TX exercis* |
| S13 | TX exercise therap* |
| S14 | TX physical activit* |
| S15 | TX muscle stretching exercis* |
| S16 | TX physical conditioning |
| S17 | TX training |
| S18 | TX walking |
| S19 | TX stretch* |
| S20 | TX strengthening |
| S21 | TX muscle strength |
| S22 | MH physical therapy modalities |
| S23 | TX physical therapy |
| S24 | TX physiotherapy |
| S25 | TX manipulation* |
| S26 | TX Workplace exercise intervention |
| S27 | TX practice |
| S28 | TX mobilization |
| S29 | S11 OR ~ S28 |
| S30 | (TI randomized) |
| S31 | (AB randomized) |
| S32 | (TI randomised) |
| S33 | (AB randomised) |
| S34 | (TI randomly) |
| S35 | (AB randomly) |
| S36 | S30 OR ~ S35 |
| S37 | S10 AND S29 AND S36 |
|  | Number of records: 3842  Date: March 31, 2025 |

| **Web of Science** | |
| --- | --- |
| #1 | ALL=(“back pain”) OR ALL=(“back pain without radiation”) OR ALL= (“low back pain”) OR ALL=("lumbago")OR ALL=(“low back pain”) OR ALL=(“LBP”) OR ALL=(Low Back Ache) |
| #2 | ALL=(exercise) OR ALL=exercis* OR ALL=(“exercise therap*") OR ALL=( "physical activit*") OR ALL=( "muscle stretching exercis*" ) OR ALL=( "physical conditioning") OR ALL= training OR ALL= walking OR ALL=stretch* OR ALL= strengthening OR ALL= ("muscle strength") OR ALL=("physical therapy modalities") OR ALL=( "physical therapy") OR ALL= physiotherapy OR ALL=manipulation* OR ALL=("Workplace exercise intervention") OR ALL="practice" OR ALL="mobilization" |
| #3 | TS=randomized OR TS=randomised OR TS=randomly |
| #4 | #1 and #2 and #3 |
|  | Number of records: 7787  Date: March 31, 2025 |

| **Ichushi-web** | |
| --- | --- |
| 背痛/TH | or 背部痛/AL |
|  | or 背痛/AL |
| 腰痛/TH | or 腰痛/AL |
|  | or 腰部痛/AL |
| AND | |
| 運動/AL |  |
| 運動療法/TH | or 機能訓練/AL |
|  | or 機能療法/AL |
| ストレッチング/TH | or ストレッチ/AL |
|  | or ストレッチ体操/AL |
| 歩行訓練/TH | or 歩行/AL |
| レジスタンストレーニング/TH | or ウェートトレーニング/AL |
|  | or スクワット･トレーニング/AL |
|  | or ストレングストレーニング/AL |
|  | or プッシュアップ/AL |
|  | or レジスタンスエクササイズ/AL |
|  | or レジスタンス・トレーニング/AL |
|  | or 筋トレーニング/AL |
|  | or 筋強化訓練/AL |
|  | or 筋力トレーニング/AL |
|  | or 筋力強化訓練/AL |
|  | or 筋力訓練/AL |
|  | or 抵抗運動トレーニング/AL |
| 活動量計/TH | or フィットネストラッカー/AL |
|  | or 身体活動量計/AL |
| 関節授動術/TH | or 可動化療法/AL |
|  | or 関節モビライゼーション/AL |
|  | or 関節モビリゼーション/AL |
|  | or 関節受動術/AL |
|  | or 関節授動/AL |
|  | or 関節可動域訓練/AL |
| 理学療法/TH | or 理学療法/AL |
|  | or マニピュレーション/AL |
| AND | |
| ランダム化比較試験/TH | or 無作為/AL |
|  | or ランダム/AL |
| Number of records: 335  Date: March 31, 2025 | |

**Supplementary Table 2.** Characteristics of the included studies.

|  |  | Kim, 2014 | Phattharasupharerk, 2019 | Shariat, 2019 | | | Almhdawi, 2020 |
| --- | --- | --- | --- | --- | --- | --- | --- |
| Participants | All | 74 | 72 | 76 | | | 41 |
|  | IG | 37 | 36 | Group 1: 19 | Group 2: 19 | Group 3: 19 | 21 |
|  | CG | 37 | 36 | Group 4: 19 | | | 20 |
| Age,  Mean (SD) | IG | 29.7 (3.9) (n = 27) | 35.7 (3.6) |  |  |  | 40.48 (7.22) |
|  | CG | 28.6 (3.2) (n = 26) | 34.8 (4.3) |  |  |  | 41.70 (6.35) |
| Sex,  Male / Female | IG | 0 / 37 | 12 / 14 |  |  |  | 7/14 |
|  | CG | 0 / 37 | 14 / 22 |  |  |  | 12/8 |
| During the symptoms |  | ≥3 months | ≥12 weeks | ≥3 months | | | ≥3 months |
| Intervention | IG | 1. CORE exercise program at home (30 min, 5 times per week, for 8 weeks）  2. Standard treatment including transcutaneous electrical nerve stimulation (20 min) and hot-pack treatment (15 min) | 1. Qigong practice session at workstation (1 h per week, 6 weeks) 2. Qigong exercise at home everyday | Group 1. Exercise therapy (40–45 min per week for 6 weeks) | Group 2. Psychotherapy (40–45 min per week for 6 weeks) | Group 3. Exercise therapy followed by relaxation therapy (40–45 min per week for 6 weeks) | 1. Use of smartphone application “Relieve my back” for 6 weeks  2. General advice, office-based stretching exercises, and home-based strengthening exercises for lower back and abdominal muscles |
|  | CG | Standard treatment including transcutaneous electrical nerve stimulation (20 min) and hot-pack treatment (15 min) | General advice on managing low back pain | No intervention | | | Placebo application and general nutrition advice |
| Duration |  | 8 weeks | 6 weeks | 6 weeks | | | 6 weeks |
| Outcome |  | VAS at rest and during movement Pain pressure threshold Lumbar active ROM Proprioceptive acuity | VAS RMDQ Back ROM Core stabilizer muscle strength Heart rate Respiration in sitting position ST-5 GPE | Pain severity based on the FRI QoL questionnaire HADS | | | VAS ODI SF-12 PSQI IPAQ |
| Assessment period |  | 8 weeks 2 months after intervention | 1–6 weeks | 6 weeks 12 weeks | | | 6 weeks 12 weeks |

**Abbreviations.** IG, intervention group; CG, control group; SD, standard deviation; VAS, visual analog scale; RMDQ, Roland-Morris disability questionnaire; ROM, range of motion; ST-5, Srithanya stress scale; GPE, global perceived effect; FRI, functional rating index; QoL, quality of life; HADS, hospital anxiety and depression; ODI, Oswestry disability index; SF-12, MOS 12-item short-form health survey; PSOI, Pittsburgh sleep quality index; IPAQ, international physical activity questionnaire.

**Supplementary Table 3.** Results of RoB 2 for each outcome.

| Outcome | | Study | Risk of bias domain | | | | | |
| --- | --- | --- | --- | --- | --- | --- | --- | --- |
|  |  |  | 1 | 2 | 3 | 4 | 5 | Overall |
| Pain intensity | VAS | Kim, 2014 | Low | High | High | High | Some concerns | High |
|  |  | Phattharasupharerk, 2019 | Low | Low | Low | High | Some concerns | Some concerns |
|  |  | Almhdawi, 2020 | Some concerns | Low | Low | Low | Low | Low |
|  | FRI | Shariat, 2019 | Some concerns | High | Low | High | Some concerns | High |
| Functional disability | RMDQ | Phattharasupharerk, 2019 | Low | Low | Low | High | Some concerns | Some concerns |
|  | ODI | Almhdawi, 2020 | Some concerns | Low | Low | Low | Low | Low |
| Physical activity | IPAQ | Almhdawi, 2020 | Some concerns | Low | Low | Low | Low | Low |

Abbreviations. VAS, visual analog scale; FRI, functional rating index; RMDQ, Roland-Morris disability questionnaire; ODI, Oswestry disability index; IPAQ, international physical activity questionnaire.

**Supplementary Table 4.** Summary of certainty of evidence results for each outcome.

|  | No of studies | No of participants | Risk of bias | Inconsistency | Imprecision | Indirectness | Publication bias | Effect | 95% CI | Certainty |
| --- | --- | --- | --- | --- | --- | --- | --- | --- | --- | --- |
| Pain intensity  (short-term） | 3 | 166 | serious ^a^ | not serious | serious ^c^ | not serious | strongly suspected ^g^ | −39.02 | −44.18 to −33.86 | VERY LOW |
| Functional disability (short-term) | 2 | 113 | serious ^b^ | not serious | very serious ^d, e^ | not serious | strongly suspected ^g^ | RMDQ | | VERY LOW |
|  |  |  |  |  |  |  |  | −2.00 | −3.57 to −0.43 |  |
|  |  |  |  |  |  |  |  | ODI | |  |
|  |  |  |  |  |  |  |  | −10.47 | −16.57 to −4.37 |  |
| Physical activity  (short-term) | 1 | 41 | not serious | serious ^c^ | very serious ^d, f^ | not serious | strongly suspected ^g^ | −1635.85 | −3876.07 to 604.37 | VERY  LOW |

a, one included study was judged as having “high” risk of bias; b, one included study was judged as having “some concerns”; c, not possible to assess heterogeneity; d, small sample size (< 400); e, not possible to assess the accuracy of integrated data; f, no statistically significant difference; g, possibility of publication bias.

Abbreviations. CI, confidence interval; RMDQ, Roland-Morris disability questionnaire; ODI, Oswestry disability index.
